# Lipidomic Analysis Reveals Specific Differences between Fibroblast and Keratinocyte Ceramide Profile of Patients with Psoriasis Vulgaris

**DOI:** 10.3390/molecules25030630

**Published:** 2020-01-31

**Authors:** Wojciech Łuczaj, Adam Wroński, Pedro Domingues, M Rosário Domingues, Elżbieta Skrzydlewska

**Affiliations:** 1 Department of Analytical Chemistry, Medical University of Bialystok, Mickiewicza 2d, 15-222 Bialystok, Poland; elzbieta.skrzydlewska@umb.edu.pl; 2 Dermatological Specialized Center “DERMAL” NZOZ in Bialystok, 15-453 Bialystok, Poland; adam.wronski@dermal.pl; 3 Mass Spectrometry Center, QOPNA, Department of Chemistry, University of Aveiro, Campus Universitário de Santiago, 3810-193 Aveiro, Portugal; p.domingues@ua.pt (P.D.); mrd@ua.pt (M.R.D.); 4 Department of Chemistry & CESAM & ECOMARE, University of Aveiro, Campus Universitário de Santiago, 3810-193 Aveiro, Portugal

**Keywords:** ceramides, psoriasis vulgaris, lipidomics, keratinocytes, fibroblasts, skin

## Abstract

Ceramides are important lipid metabolites for primal skin functions. There is increasing evidence that alteration of the profile and metabolism of ceramides is associated with skin diseases, such as psoriasis vulgaris. Most studies have reported alteration in ceramide content in the stratum corneum, but these have been scarcely reported for other skin layers. In the present work, we aimed to explore changes in the ceramide profile of fibroblasts and keratinocytes in patients with psoriasis vulgaris and healthy subjects. Using the reversed-phase liquid chromatography-quadrupole-time-of-flight-tandem-mass spectrometry (RPLC-QTOF-MS/MS) platform, we identified ceramide containing non-hydroxy fatty acid ([N]), α-hydroxy fatty acid ([A]), and esterified ω-hydroxy fatty acid ([EO]) and 3 sphingoid bases, dihydrosphingosine ([DS]), sphingosine ([S]), and phytosphingosine ([P]). We found that in the keratinocytes of patients with psoriasis, CER[NS], CER[NP], CER[AS], CER[ADS], CER[AP] and CER[EOS] tended to be expressed at higher relative levels, whereas CER[NDS] tended to be expressed with lower levels than in healthy subjects. In the case of fibroblasts, significant differences were observed, mainly in the three ceramide classes (CER[AS], CER[ADS] and CER[EOS]), which were expressed at significantly higher levels in patients with psoriasis. The most significant alteration in the fibroblasts involved elevated levels of CER[EOS] that contained ester-linked fatty acids. Our findings provide insights into the ceramide profile in the dermis and epidermis of patients with psoriasis and contribute for the research in this field, focusing on the role of keratinocyte-fibroblast crosstalk in the development of psoriasis vulgaris.

## 1. Introduction

Psoriasis is a chronic inflammatory skin disease that affects men and women of all ages. It is one of the most common skin disease, and the characteristic symptoms are psoriatic plaques that involves patches of rough, red skin and silvery-white scales [1]. Although it primarily affects the skin, it is usually associated with systemic impact, affecting other organs, and causing other morbidities, such as rheumatoid arthritis and cardiovascular diseases [1]. It is a debilitating disease which has a great negative impact on the quality of life of patients [1]. However, the pathophysiology of this disease is not completely understood, and the treatment is only based on symptomatic relief.

The skin, which is largely affected in psoriasis, is the largest organ of the body and comprises three layers: epidermis, dermis and hypodermis. The epidermis is the outer layer of the skin, comprising mainly 95% differentiated keratinocytes, and its outermost layer is the stratum corneum, which is composed of corneocytes (terminally differentiated keratinocytes) and mortar lipid structure [2,3]. The dermis is the middle layer where collagen, elastin, vascular and nerves are the main constituents of the matrix in which fibroblasts are the predominant cells. Under the dermis is the hypodermis, a layer composed of adipocytes that are essential for metabolism and energy storage [2]. 

Most of the studies on psoriasis reported alteration in the stratum corneum and epidermal layer [4,5,6], with changes in the keratinocytes [7]. However, the alterations of other skin and epidermal layers have not been addressed and the crosstalk and signal transduction between the epidermal and dermal layers may have significant roles in the health and disease of the skin **[2]**. Although the pathogenesis of this disease is not fully clarified, it is widely accepted that there is an association of psoriasis with the immune system increased activity and the overexpression of inflammatory agents [8]. Inflammation and immune response are regulated by lipids and their metabolites [9]. Furthermore, changes in lipid composition of skin cell membranes were reported during inflammatory processes in the skin, leading to various skin lesions [10]. Recently, an untargeted lipidomics approach has been used to investigate the alteration of lipid metabolites in psoriasis, to provide novel insights into the role of lipids in this skin disease. The metabolism of phospholipids, such as lysophosphatidic acid, lysophosphatidylcholines, phosphatidic acid, phosphatidylinositols, and phosphatidylcholines, was significantly altered in the plasma of psoriatic patients [11]. Moreover, it has been reported changes in the ceramide profile in the stratum corneum of patients with psoriasis [6]. 

**Figure 1:**
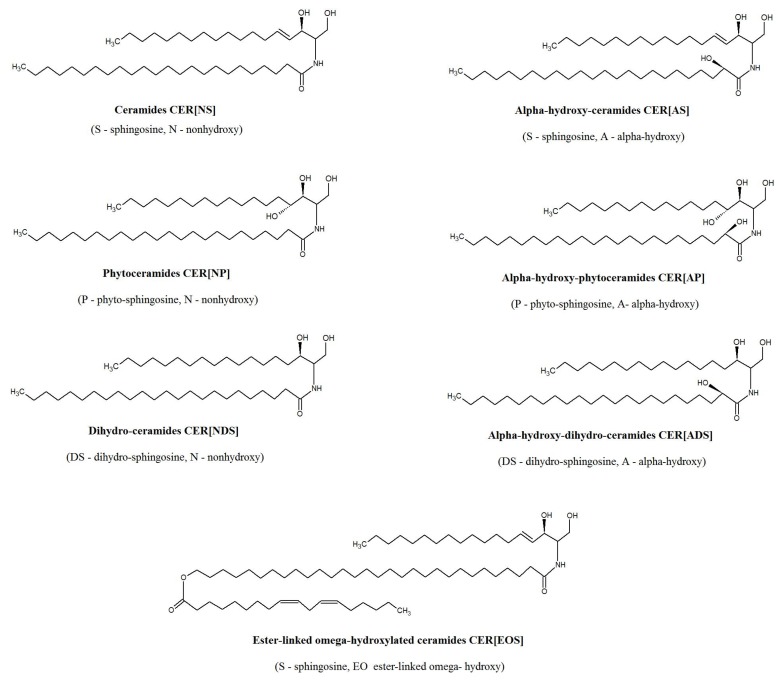
Major subclasses of ceramides present in epidermis.

Ceramides are considered to be the most important lipid metabolites regarding primal skin functions [12]. Within the structure of the epidermal membrane, ceramides are the dominant lipid class by weight (50%) and exhibit the greatest molecular heterogeneity, comprising at least 11 classes [13]. Thus, a nomenclature using combination of abbreviations for types of sphingoid bases (dihydrosphingosine [DS], sphingosine [S], and phytosphingosine [P]) and fatty acids (non-hydroxy fatty acid [N], α-hydroxy fatty acid [A], and esterified ω-hydroxy fatty acid [EO]) has been proposed to discriminate the epidermal ceramide classes (Figure 1). The symbols in brackets indicate the notation of each block, according to the nomenclature used by Masukawa et al. [14]. It has been shown that human stratum corneum is composed of CER[NP] (22.1%), CER[NH] (14.5%), CER[AH] (10.8%), CER[NDS] (9.8%), CER[AS] (9.6%), CER[AP] (8.8%), CER[NS] (7.4%), CER[EOS] (6.5%), CER[EOH] (4.3%), CER[ADS] (1.6%), CER[EOP] (1.1%), and CER[EODS] (0.4%) [5]. Ceramides play a crucial role in the formation and maintenance of the integrity of the skin barrier [15]. In general, ceramides containing esterified fatty acids are more specific to skin and are important for epidermal barrier formation [16]. Moreover, ceramides and their metabolites have been implicated in cell signaling and linked to cell proliferation, differentiation and apoptosis in the human epidermis [17]. There are also several studies indicating an association of ceramides with skin diseases, including psoriasis. It has been shown that CER[EOS], CER[NP], and CER[AP] levels were decreased in psoriatic patients [4]. Moreover, atopic dermatitis patients exhibited decreased level of CER[EOS], CER[EOH], CER[EOP], CER[NH], and CER[NP] among the stratum corneum ceramide classes [18]. However, the majority of these studies focused on the characterization of the ceramide profile in stratum corneum [4,7,18]. The analysis of the stratum corneum allows investigating surface skin ceramides but does not provide information on how psoriasis affects the other layers of the epidermis and dermis, nor on the role of the crosstalk between the main cellular constituents, respectively keratinocyte and fibroblast, in the development of psoriasis vulgaris. Therefore, in the present study, we aimed to extend this knowledge and examined the changes in the ceramide profile of the two types of skin cells, fibroblasts and keratinocytes, of patients with psoriasis vulgaris compared to healthy subjects, using modern lipidomics approaches based on LC-MS.

## 2. Results

We used a lipidomics approach to characterize the keratinocyte and fibroblast ceramide profile of healthy subjects and patients with psoriasis vulgaris. We identified seven types of ceramides (Table 1).

Using the RPLC-QTOF-MS/MS platform, we identified more than 50 ceramide species belonging to the following classes: CER[NS], CER[NDS], CER[NP], CER[ADS], CER[AS], CER[AP], CER[EOS] (Figures S1–S3 and Tables S1–S3 and Supplementary Materials). 

**Figure 2:**
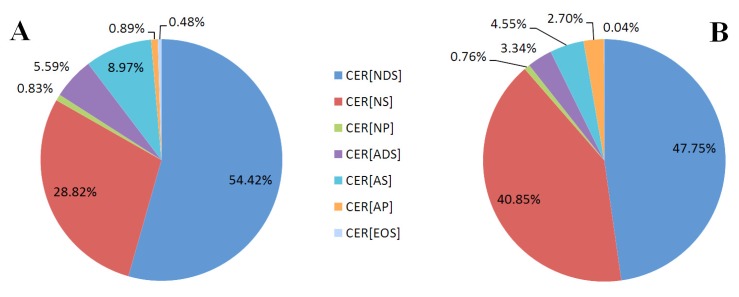
The proportions of ceramide families in the keratinocytes (**A**) and fibroblasts (**B**) of healthy subjects; (non-hydroxy fatty acid [N], α-hydroxy fatty acid [A], and esterified ω-hydroxy fatty acid [EO], dihydrosphingosine [DS], sphingosine [S], and phytosphingosine [P]).

We found that the proportions of ceramide classes were different in the two types of skin cells (Figure 2). The differences mainly concerned the CER[NDS], CER[NS] and CER[AS] classes. Keratinocytes had a higher relative content of CER[NDS] and CER[AS], while fibroblasts had a higher relative abundance of the class of CER[NS] and CER[AP] than keratinocytes. In both cells, CER[NDS] and CER[NS] were the most abundant classes. 

**Figure 3:**
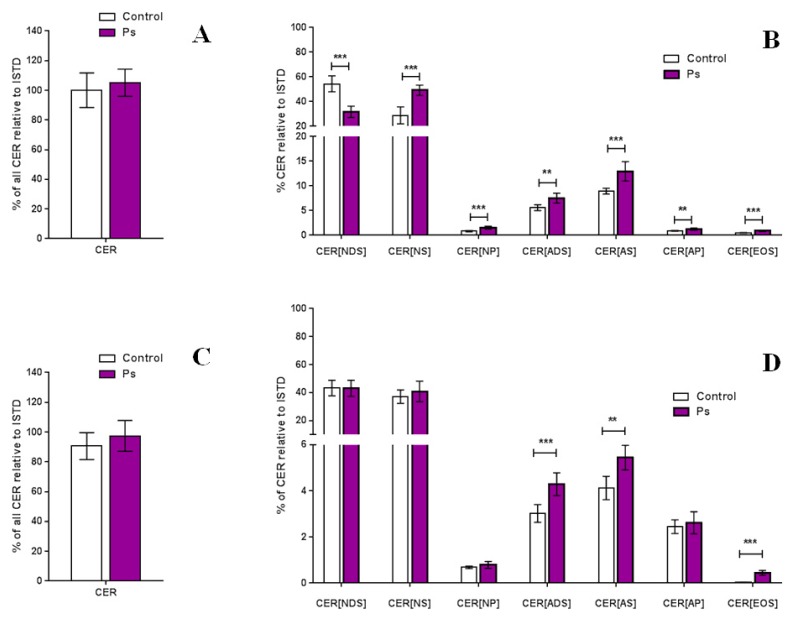
Total amount of ceramides in the keratinocytes (**A**) and fibroblasts (**C**) and relative content of each ceramide class in the keratinocytes (**B**) and fibroblasts (**D**) of healthy subjects and patients with psoriasis vulgaris (Ps); (values are mean ± SD *** p < 0.001; ** p < 0.01); *(non-hydroxy fatty acid [N], α-hydroxy fatty acid [A], and esterified ω-hydroxy fatty acid [EO], dihydrosphingosine [DS], sphingosine [S], and phytosphingosine [P], internal standard [ISTD]).*

The total amount of ceramides and percentage composition of the ceramide classes is shown in Figure 3. After comparing the variation in the content for each class in the control and disease samples, no significant changes were observed in the total relative amount of ceramides in both types of skin cells. However, significant differences were detected by separating ceramides by classes.

By comparing the relative content of each ceramide class, we found significant changes in all CER classes in keratinocytes, obtained from healthy volunteers and from patients with psoriasis. The CER[NS], CER[NP], CER[AS], CER[ADS], CER[AP] and CER[EOS] tended to be expressed at higher relative levels in patients with psoriasis, while CER[NDS] tended to be expressed at a lower level than that of healthy subjects (Figure 3). In the case of fibroblasts, significant differences we observed only in three ceramide classes, namely CER[AS], CER[ADS] and CER[EOS], which are the minor ceramide classes of the skin, which levels were significantly higher in patients with psoriasis (Figure 3). Importantly, we found that the most significant alteration in the fibroblasts concerns an elevated level of CER[EOS] class that contains fatty acids linked to esters.

**Figure 4:**
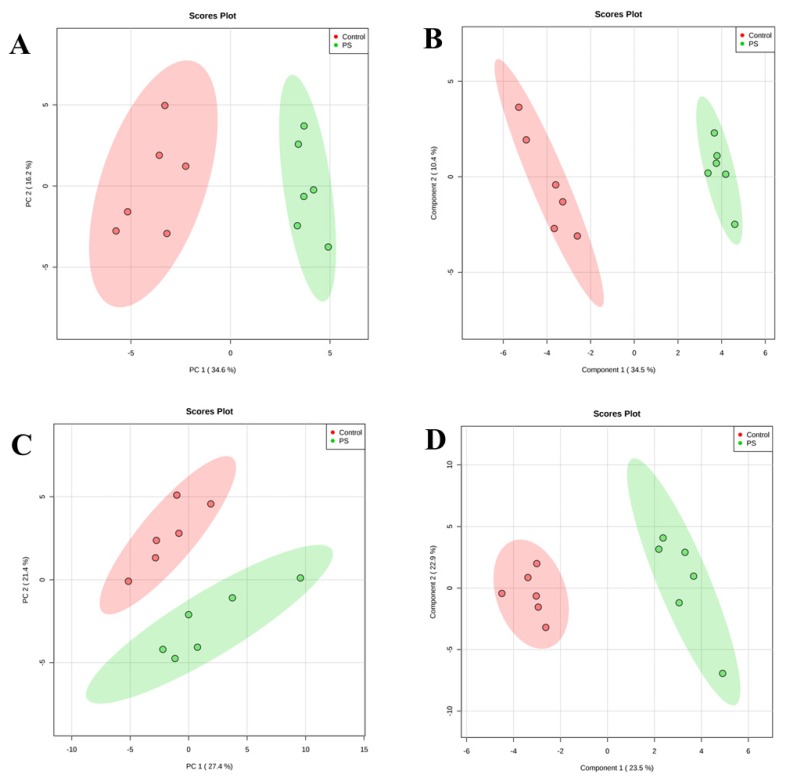
Principal component analysis (PCA) (left panels) and partial least square discriminant analysis (PLS-DA) (right panels) in a two-dimensional score plot of the relative abundance of ceramide species determined by RPLC-QTOF-MS in keratinocytes (**A,B**) and fibroblasts (**C,D**) of healthy subjects and patients with psoriasis vulgaris (Ps).

We used multivariate statistical analysis to extract the ceramide species of each class responsible for the changes observed in the CER profiles of keratinocytes and fibroblasts between healthy subjects and patients with psoriasis. The PCA models showed that the group of healthy subjects was clearly separated from the patient groups (Figure 4). Control samples were scattered over the left region of the plot, while patients were scattered across the right region of the plot. The separation was slightly better in case of the keratinocytes, where the PCA model captured 50.8% of the total variance (PC1 (34.6%), PC2 (16.2%)) (Figure 4). The variation between the biological groups in keratinocytes is more pronounced on the PC1, which is most likely associated with the state of health. The PCA model for fibroblasts captured 48.8% of the total variance (PC1 (27.4%) and PC2 (21.4%)) (Figure 4). 

Finally, we used partial least squares-discriminate analysis (PLS-DA) and VIP (variable importance in projection) to estimate the importance of each molecular species of ceramides which led to the separation of the groups. The PLS-DA model exhibited the performance statistics of R^2^ = 0.96723 for keratinocytes, R^2^ = 0.9532 for fibroblasts and a high prediction parameter Q^2^ = 0.89017 and Q^2^ = 0.7173 for keratinocytes and fibroblasts, respectively. The resulting two-dimensional PLS-DA score plot showed that both groups were well clustered in the two types of skin cells (Figure 4). The PLS-DA score plot described 44.6% of the total variance (34.5%—component 1, 10.4%—component 2) in case of keratinocytes, and 46.4% of the total variance (component 1—34.5%; component 2—10.4%) for fibroblast samples. 

The VIP lists of the 16 ceramide species with higher discriminating power for each type of skin cell are presented in Table 2 and Table 3. The majority of these relevant ceramide species identified in keratinocytes belonged to the CER[NDS] and CER[NS] classes which were found to be down-regulated and up-regulated, respectively, in the keratinocytes of patients (Table 2). However, an emerging group of ceramide species in fibroblasts involved different ceramide classes. Most of them (mainly CER[EOS], CER[AS] and CER[ADS]) were up-regulated in patients fibroblasts, while some species of CER[NDS], CER[NS] and CER[NP] had the tendency to be down-regulated (Table 3). Nevertheless, it should be noted that CER[EOS] had the highest VIP score and were on the top of the ceramide species list that characterized fibroblasts (Table 3).

**Figure 5:**
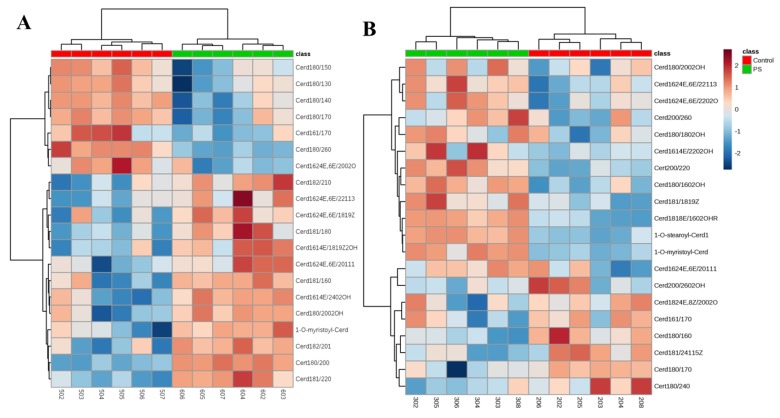
Two-dimensional hierarchical clustering heat map of the 20 CERs selected according to the VIP list in the keratinocytes (**A**) and fibroblasts (**B**). Relative abundance levels are indicated on the colour scale, with numbers indicating the fold difference from the overall average. The clustering of the sample groups is represented by the dendrogram at the top. The clustering of individual CER species with regard to their similarity in the change of relative abundance is represented by the dendrogram on the left.

We created a dendrogram with a two-dimensional hierarchical clustering, using the top 20 ceramides selected according to the VIP list (Figure 5). The primary split in the upper hierarchical dendrogram shows that the samples clustered independently into the two groups in case of both types of skin cells examined. The clustering of individual ceramides with respect to their similarity in changes of expression showed that they clustered into two principal groups. The first group consisted of ceramide species, which were more abundant in healthy subjects and the second group had ceramide species, which were less abundant in the control group.

In case of keratinocytes, the first group consisted mainly of CER[NDS], while CER[NS], CER[AS], CER[ADS] as well as certain species belonging to the CER[NP] and CER[EOS] were in the second group. However, in fibroblasts, CER[EOS], CER[AS] and CER[ADS] were less abundant in the control group, while the relative levels of some of CER species belonging to CER[NS] and CER[NDS] were higher, compared to their levels in patients with psoriasis.

## 3. Discussion

In this study, we have evaluated the adaption of the ceramide profile of keratinocytes and fibroblasts of healthy subjects and patients suffering from psoriasis vulgaris using the RPLC-QTOF-MS/MS lipidomic platform. Among the identified ceramide species, ceramide-containing odd-chain fatty acids were present. Initially, odd-chain fatty acids were considered to be derived solely from diet and exist in cell at much lower levels compared with even-numbered fatty acids [19]. However, subsequently they were recognized as a product of amino acid catabolism within mitochondria in adipose tissue. Moreover, identification of ceramides containing odd-chain fatty acids in human skin has been also reported [20]. Moreover, it has been shown that ceramides containing odd-numbered fatty acids are specific to stratum corneum where their levels were accounted for 30% of total ceramides [21]. Odd-numbered and even-numbered fatty acids, both are similarly metabolized to glycerophospholipids [22]. Thus, odd-numbered fatty acids itself might not have physiological significance. *However, recently* the positive associations of odd-chain fatty acids with cardiovascular outcomes were shown [23]. No significant change was observed in the total amount of ceramides in the two types of skin cells. However, we found significant differences in the relative amount of ceramide classes, depending on the type of cells. Our results showed higher levels of CER[NS], CER[NP], CER[AS], CER[ADS], CER[AP] and CER[EOS] in the keratinocytes of patients with psoriasis, while CER[NDS] level was lowered, when compared with the control samples. In the case of fibroblasts, changes were observed for CER[AS], CER[ADS] and CER[EOS] and significantly higher levels of their relative amounts were expressed in patients with psoriasis, compared to healthy subjects. In contrast to our results, other studies reported a decrease in the level of ceramides, e.g., CER[NP], CER[ADS], CER[AP] or CER[EOS] in the stratum corneum of patients with psoriasis [4,6,7]. However, these authors did not evaluate the variation of CERs in the other layers of skin, as this study shows. In addition, reductions in the synthesis of ceramides and their epidermal level were positively correlated with the Psoriasis Area and Severity Index (PASI) score in mild to moderate psoriasis [24,25]. Given these results, our observations may be related to an increased synthesis of CER in the dermis and epidermis, in response to a decrease in ceramide levels in stratum corneum. It is important to note that a decrease in ceramide in stratum corneum causes loss of water and dysfunction of the barrier in the epidermis, comprising loss of protection against antigens, including bacteria, and can lead to skin abnormalities such as psoriasis [14,26]. Basically, the epidermal barrier is formed by the action of lipids generated in the lamellar bodies, also known as Odland bodies, during the process of keratinocyte differentiation [16]. The major lipids that form the lamellar barrier of the skin consist of 50% ceramide [27]. It has been shown that CER[NP] and especially CER[EOS] are essential components in creating the lamellar structure [15]. Thus, it can be assumed that observed increased level of CER[NP] and CER[EOS] in the keratinocytes of patients with psoriasis may be a response to alteration of epidermal barrier. Consequently, the tendency observed of up-regulation of most of the classes of ceramides in the fibroblasts, but especially in the keratinocytes of patients with psoriasis, may be also the response to the inflammatory processes in the skin. However, the mechanisms by which the epidermal synthesis of ceramides is regulated are not fully understood, particularly at the molecular level. For example, in the epidermis, it has been described that peroxisome proliferator-activated receptors (PPARs) improve the permeability barrier by increasing the synthesis of ceramides [28]. Furthermore, PPARβ/δ has been demonstrated as a unique transcription factor modulating epidermal homeostasis due to its prominent upregulation during the transcriptional induction of genes involved in the synthesis of ceramides [29]. In general, a possible mechanism for increasing the production of ceramide is by de novo biosynthesis of ceramides, which is mainly catalyzed by serine palmitoyltransferase. However, studies on skin biopsies taken from patients with psoriasis have revealed that the level of de novo synthesis of ceramides, the expression of serine palmitoyltransferase and the number of ceramides are significantly lower in psoriatic plaques, when compared to the non-lesional epidermis, thus limited to the stratum corneum, and no relation has been found to the other layers of the epidermis. The other two additional pathways for generating ceramides are the degradation of glucosylceramide by glucosylceramide-β-glucosidase, and the hydrolysis of sphingomyelin, catalyzed by sphingomyelinase. However, psoriatic skin has been shown to have reduced glucosylceramide-β-glucosidase mRNA expression, compared to normal healthy skin, although the level of mRNA for this enzyme is higher in psoriatic plaques than in non-lesional skin [30]. In the same study, it was also shown that the level of sphingomyelinase was lower in the stratum corneum of psoriatic lesions compared to non-lesional psoriatic skin, which could justify the higher levels of ceramides observed in our study [30]. Moreover, psoriatic plaques have also been shown to have significantly lower levels of prosaposin, compared to psoriatic non-lesional skin and healthy skin. Prosaposin is a precursor of saposin, a nonenzymatic cofactor, which is necessary for the hydrolysis of sphingolipids, including sphingomyelins, and thus contributes to increasing the level of ceramides in psoriasis [30]. Our results are in agreement with both hypotheses and these can justify the observed increase in ceramides level. Other specific mechanism leading to changes in plasma membrane ceramide content involves delivery of ceramides to lysosomes, as part of the endocytic vesicles during the delivery of extracellular or membrane components (e.g., raft components) for their conversion to sphingomyelin and reutilization [31]. Ps is associated with oxidative stress and inflammation and it has been observed an increase in cellular ceramide as a result of various stimuli such as oxidative stress [32], and the initiation of the inflammatory cytokine cascade [33]. It was also shown that ceramides induce both apoptosis [34] and autophagy [35,36]. Increased stress conditions stimulate the production of ceramides by activation of sphingomyelinase, as well as by inducing de novo ceramides production. Furthermore, alkaline sphingomyelinase catalyzes the hydrolysis of sphingomyelin to lysophosphatidylcholine and platelet-activating factor (PAF) to suppress inflammatory responses, in addition to the conversion of sphingomyelin to ceramide [37]. 

Our study showed significant differences in ceramide classes between the cells of the two skin layers of patients with psoriasis and healthy subjects. These differences were detected in CER[NS] and CER[NDS] ceramides, the relative levels of which increased and decreased, respectively, in keratinocytes, while the corresponding levels of these ceramide classes were unchanged in the fibroblasts of psoriatic patients. It has been shown that ceramide is critical for exosome formation and increased ceramides generation led to induction of exosome secretion [38]. Exosomes are cell-derived membrane vesicles that are secreted by cells in order to remove proteins and lipids, and release them in the extracellular space [39]. The role for extracellular vesicles including exosomes in the cell-to-cell communication, both in the health and in the disease has been widely described in the literature [40,41]. Thus, observed differences in ceramide levels between keratinocytes and fibroblast may by associated with different signaling functions of ceramide species in the dermis and epidermis and indicate a role of keratinocyte-fibroblast cross-talk in the development of psoriasis. Additionally, the different responses of the dermis and epidermis reported in our study could indicate compartmental differences in lipid metabolism. Regarding the observed changes, it should be noted that CER[NDS] ceramides are produced upstream of other ceramides, and initially converted into CER[NS] species, which may explain the observed changes. CER[NDS] contains sphinganine, and the higher level associated with the increased expression of ceramidase, which is the major enzyme involved in ceramides degradation, in the psoriatic epidermis was positively correlated with the clinical severity of psoriasis [42]. Furthermore, the involvement of sphingomyelinase is a possible explanation for our findings observed in keratinocytes and fibroblasts. Increased sphingomyelinase activity would result in an increased release of ceramides stored in membrane sphingomyelins. However, sphingomyelinase releases both CER[NS] and CER[AS] ceramides from sphingomyelins, at least in the stratum corneum [43], and CER[AS] were increased in keratinocytes and fibroblasts, while higher levels of CER[NS] were observed only in keratinocytes. Although CER[NS] have been identified as components of glucosylceramides in the epidermis, CER[NDS] ceramides have not been identified [44]. 

In addition to the changes mentioned, we have also observed a significant increase in the relative levels of two phytoceramide classes, namely CER[NP] and CER[AP] in keratinocytes but not in fibroblasts of patients with psoriasis. Moreover, the esterified ceramides CER[EOS] were the most relevant CER species in fibroblasts that discriminate patients with psoriasis from healthy subjects. CER[EOS], which contains long-chain fatty acids, is known to be important for the barrier function of the epidermis [45]. However, contrary to our findings, it was described that psoriatic plaques formed in stratum corneum had lower levels, not only of CER[EOS] but also of ceramides containing phytosphingosine with a concomitant higher concentration of ceramides containing sphingosine, compared to the normal healthy skin [46]. 

In conclusion, our study showed changes in the profile of ceramides in keratinocytes and fibroblasts in psoriatic patients and also between psoriatic patients and healthy subjects. Since the decrease in epidermal ceramide content has been linked to water loss and barrier dysfunction, these results draw attention to the research on the role of higher cell concentrations of ceramides and the possibility of particular ceramide species playing signaling functions in the dermis and epidermis. Furthermore, our results provide an overview of the metabolism of ceramides in the dermis and epidermis of psoriatic patients and may help to better understand the role of keratinocyte-fibroblast cross-talk in the development of psoriasis.

## 4. Materials and Methods 

### 4.1. Chemicals 

All chemicals were purchased from Sigma-Aldrich (Steinheim, Germany) and had purities greater than 95%. Ceramide internal standards [N-stearoyl 4-hydroxysphinganine, *N*-(2′-(R)-hydroxystearoyl)-d-*erythro*-sphingosine, *N*-lignoceroyl-d-erythro-sphingosine and *N*-lignoceroyl-d-erythro-sphinganine] were purchased from Avanti Polar Lipids (Alabaster, AL, USA). All solvents used were of LC-MS grade.

### 4.2. Collection of Skin Samples

Skin tissues were collected from 6 untreated patients with a diagnosis of psoriasis vulgaris (3 men and 3 women; age range 28–57 years, mean 42; these patients had the most characteristic psoriatic skin lesions from a cohort 30 selected patients) and 6 healthy volunteers (sex- and age-matched individuals forming a control group; age range 24–56 years, mean 41). Eligible patients were those who were given a diagnosis of plaque psoriasis for, at least, 6 months with, at least, 10% of the total body surface area affected. The severity of psoriasis was assessed using the Psoriasis Area and Severity Index (PASI) score (median 17; range 10–25). None of the patients or healthy subjects had received topical, injectable or oral medications during the 4 weeks before the study. Individuals whose history indicated any other disorders were excluded from the study. None of the participants were smokers. The study was approved by the Local Bioethics Committee Medical University of Bialystok (Poland), No. R-I-002/289/2017. Written informed consent was obtained from all the patients.

Skin fragments immediately after biopsy were destined for histopathological examination (hematoxylin-eosin staining). The remaining sample was washed in PBS with 50 U/mL penicillin and 50 μg/mL streptomycin and incubated overnight at 4 °C in 1 mg/mL dispase to separate epidermis from the dermis. Following incubation, the epidermis was digested using Trypsin/EDTA (20 min incubation at 37 °C). Isolated keratinocytes were washed and resuspended in PBS. The dermis was fragmented and seeded into plates for fibroblasts migration and proliferation. The growth medium was Dulbecco’s Modified Eagle Medium (DMEM) supplemented with 10% fetal bovine serum (FBS), 50 U/mL penicillin and 50 μg/mL streptomycin. Cells were cultured in a humidified atmosphere of 5% CO_2_ at 37 °C. When the cells (passage 3) reached 70% confluence, they were washed and resuspended in PBS. Separated keratinocytes and fibroblasts were lysed by sonification on ice.

### 4.3. Lipid Extraction

To obtain lipid extracts rich in ceramides, 5 mL of methanol was added to skin cells lysates fortified with 10 μL of a solution containing CER internal standards at a final concentration of 1 ng/mL, vortexed, and sonicated for 10 min. After sonication, samples were centrifuged (Thermo Scientific Sorvall Legend X1R, Pittsburgh, PA, USA) at 5000× g for 10 min at room temperature. The methanol fraction was dried at 37 °C under a nitrogen stream. The dried extract was then reconstituted in 300 μL of 11/1 hexane/isopropanol (v/v) and solid-phase extraction (SPE) was performed using NH_2_ SPE columns (100 mg, 1.0 mL from Cronus, Deerfield, IL, USA). SPE columns were preconditioned with 2 mL of hexane. After the sample load, the cartridge was washed with 2 mL of hexane and ceramides were eluted using 2 mL of a hexane/methanol/chloroform 80/10/10 (v/v) mixture. The eluted fraction was dried under nitrogen and dissolved in 300 μL of isopropanol/chloroform 50/50 (v/v) prior to LC-MS/MS analysis.

### 4.4. LC-MS/MS Analysis 

The ceramide rich extracts were analyzed by LC-MS/MS using reversed-phase (RP) chromatography to characterize the CER profiles. A 1290 Ultra high-performance liquid chromatography (UPLC) system (Agilent 1290; Agilent Technologies, Santa Clara, CA, USA) coupled to a quadrupole time of flight mass spectrometer (QTOF) (Agilent 6540; Agilent Technologies, Santa Clara, CA, USA) equipped with a Dual Jetstream ESI source was used for analysis. Ceramides were separated on an Acquity BEH Shield RP C18 column (2.1 × 100 mm; 1.7 μm; Waters, Milford, MA, USA) at 70 °C. The mobile phase consisted of 20 mM ammonium formate pH 5 (A) and methanol (B). The solvent gradient was programmed as follows: the gradient started with 70% of B held isocratically for 1 min, and linearly increased to 100% over 75 min, and returning to the initial conditions over 5 min. The flow rate through the column was 0.5 mL/min. The QTOF was operated in positive ion electrospray mode. Electrospray voltage was optimized to 3.5 kV; the drying and sheath gas temperatures were set to 300 °C, and the drying and sheath gas flow rates were set to 6 and 8 L/min, respectively. Data were collected in profile mode at an acquisition rate of 3 spectra/s in the extended dynamic range mode (2 GHz). MS/MS experiments were performed in a data-dependent acquisition mode (DDA) with an isolation width of ~1.3 Da. Product ion scan spectra were acquired in the range of *m*/*z* 100–1500, and the collision energy was fixed at 35 eV. Data acquisition was carried out with Mass Hunter data software version B0.6.0 (Agilent Technologies, Santa Clara, CA, USA). The relative abundance of each ion was calculated by normalizing the area of each extracted ion chromatogram peak to the area of an internal standard. 

### 4.5. Ceramides Identification

All ceramide species were identified by the presence of the molecular ion, [M + H]^+^ ion, retention time and typical fragmentation patterns observed in the MS/MS spectra. The typical fragmentation pattern of ceramide species includes the loss of water and fatty acid molecules, leading to the formation of characteristic product ions (Table S1, Figures S2 and S3 in the Supplementary Materials).

### 4.6. Data Treatment and Statistical Analysis

The relative ion abundances of the two groups of skin cells extracts (Ps and healthy subjects) were obtained after the LC-MS experiments. Peak areas were then corrected using the area of the selected internal standard by exporting the integrated peak area values into an excel spreadsheet (Excel, Microsoft, Redmond, WA). Data preprocessing, including baseline correction, peak alignment, and normalization was performed using MZmine 2.2 [47]. The processed data were analyzed for statistical significance using t-Test with Benjamin-Hochberg post hoc correction (*P* values) used to determine significant differences between samples. Differences were considered significant if *p* < 0.05. The data set was then subjected to multivariate analysis. The unsupervised segregation was conducted via principal components analysis (PCA) using auto-scaled data. Subsequently, a partial least squares-discriminate analysis (PLS-DA) model was constructed and variable importance in projection (VIP) scores were calculated to estimate the importance of each variable in the PLS-DA projection. The heatmap for significant ceramide species was then constructed. All the statistics approaches were performed using MetaboAnalyst version 4.0 [48].

## Figures and Tables

**Table 1:** Subclasses of ceramides identified in human fibroblasts and keratinocytes according to the nomenclature proposed by Masukawa [14].

CER Subclass	Fatty Acid	Sphingoid Base
CER[NDS]	non-hydroxy [N]	dihydrosphingosine [DS]
CER[NS]	non-hydroxy [N]	sphingosine [S]
CER[NP]	non-hydroxy [N]	phytosphingosine [P]
CER[ADS]	α-hydroxy [A]	dihydrosphingosine [DS]
CER[AS]	α-hydroxy [A]	sphingosine [S]
CER[AP]	α-hydroxy [A]	phytosphingosine [P]
CER[EOS]	esterified ω-hydroxy [EO]	sphingosine [S]

**Table 2:** Changes in 16 ceramides with higher discriminating power identified in keratinocytes with variable importance in projection (VIP) score greater than one; the colored circles indicate the relative level of the corresponding metabolite in each group under study.

m/z	RT	VIP Score	CER Species	Class	Control	Ps
612.5858	42.11	1.71	Cer(t18:0/20:0)	CER[NP]	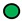	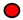
680.6881	34.91	1.65	Cer(d18:0/26:0)	CER[NDS]	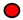	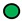
622.6056	34.91	1.57	Cer(d18:1/22:0)	CER[NS]	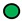	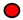
512.4985	32.79	1.40	Cer(d18:0/14:0)	CER[NDS]	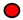	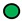
590.5429	38.94	1.38	Cer(d18:2/20:1)	CER[NS]	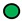	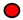
566.5429	36.95	1.37	Cer(d18:1/18:0)	CER[NS]	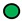	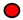
638.5996	43.02	1.35	Cer(d16:1/24:0(2OH))	CER[AS]	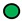	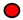
554.5423	40.60	1.34	Cer(d18:0/17:0)	CER[NDS]	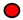	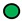
552.4898	41.10	1.32	Cer(d16:1/18:1 (2OH))	CER[AS]	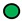	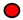
526.5142	36.49	1.32	Cer(d18:0/15:0)	CER[NDS]	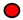	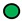
538.5148	32.43	1.30	Cer(d18:1/16:0)	CER[NS]	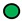	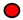
606.5752	35.22	1.29	Cer(d18:2/21:0)	CER[NS]	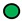	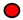
612.5870	41.46	1.29	Cer(d18:0/20:0(2OH))	CER[ADS]	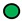	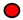
748.7098	64.50	1.28	1-O-myristoyl-Cer(d18:1/16:0)	CER[EOS]	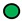	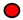
580.5255	33.33	1.27	Cer(d16:2/20:0(2OH))	CER[AS]	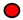	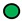
524.4978	33.56	1.23	Cer(d16:1/17:0)	CER[NS]	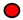	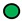

Ceramide (CER), non-hydroxy fatty acid [N], α-hydroxy fatty acid [A], and esterified ω-hydroxy fatty acid [EO], dihydrosphingosine [DS], sphingosine [S], and phytosphingosine [P], retention time (RT). Low: 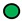; high: 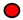.

**Table 3:** Changes in 16 ceramides with higher discriminating power identified in fibroblasts with variable importance in projection (VIP) score greater than one, along with their respective changes; the colored circles indicate the relative level of the corresponding metabolite in each group under study.

m/z	RT	VIP Score	CER Species	Class	Control	Ps
612.5858	42.11	1.71	Cer(t18:0/20:0)	CER[NP]	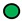	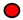
680.6881	34.91	1.65	Cer(d18:0/26:0)	CER[NDS]	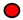	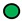
622.6056	34.91	1.57	Cer(d18:1/22:0)	CER[NS]	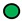	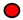
512.4985	32.79	1.40	Cer(d18:0/14:0)	CER[NDS]	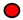	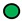
590.5429	38.94	1.38	Cer(d18:2/20:1)	CER[NS]	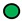	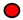
566.5429	36.95	1.37	Cer(d18:1/18:0)	CER[NS]	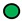	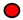
638.5996	43.02	1.35	Cer(d16:1/24:0(2OH))	CER[AS]	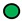	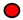
554.5423	40.60	1.34	Cer(d18:0/17:0)	CER[NDS]	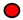	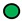
552.4898	41.10	1.32	Cer(d16:1/18:1 (2OH))	CER[AS]	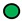	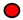
526.5142	36.49	1.32	Cer(d18:0/15:0)	CER[NDS]	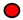	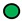
538.5148	32.43	1.30	Cer(d18:1/16:0)	CER[NS]	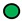	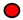
606.5752	35.22	1.29	Cer(d18:2/21:0)	CER[NS]	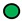	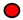
612.5870	41.46	1.29	Cer(d18:0/20:0(2OH))	CER[ADS]	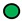	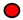
748.7098	64.50	1.28	1-O-myristoyl-Cer(d18:1/16:0)	CER[EOS]	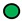	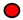
580.5255	33.33	1.27	Cer(d16:2/20:0(2OH))	CER[AS]	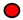	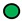
524.4978	33.56	1.23	Cer(d16:1/17:0)	CER[NS]	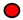	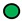

Ceramide (CER), non-hydroxy fatty acid [N], α-hydroxy fatty acid [A], and esterified ω-hydroxy fatty acid [EO], dihydrosphingosine [DS], sphingosine [S], and phytosphingosine [P], retention time (RT). Low: 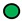; high: 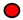.

## Supplementary Materials

Table S1. MS-based identification of the ceramide molecular species quantified in the present study, Table S2. Peak area of each ceramide molecular species identified in the keratinocytes obtained using MZmine software (XLSX), Table S3. Peak area of each ceramide molecular species identified in the fibroblasts obtained using MZmine software (XLSX), Figure S1. An example of the total ion chromatogram (TIC), Figure S2. And Figure S3. Examples of CER specie identification.
