# Supplementary material for: Lipidomic Analysis Reveals Specific Differences between Fibroblast and Keratinocyte Ceramide Profile of Patients with Psoriasis Vulgaris
Source: Molecules. 2020 Jan 31;25(3):630. doi: 10.3390/molecules25030630 (PMC7037443; doi:10.3390/molecules25030630)
Supplement: Supplementary file 1 [file molecules-25-00630-s001.zip › molecules-700047-SM - revised.docx]

**Table S1.** MS-based identification of the ceramide molecular species quantified in the present study. m/z values of characteristic fragments observed on MSMS spectra have been given. *(non-hydroxy fatty acid [N], α-hydroxy fatty acid [A], and esterified ω-hydroxy fatty acid [EO], dihydrosphingosine [DS], sphingosine [S], and phytosphingosine [P]; fatty acid [FA])*

| **CER Class** |  | **CER specie** | |  | **m/z of characteristic fragments on MS/MS spectra** | | | |
| --- | --- | --- | --- | --- | --- | --- | --- | --- |
|  | **m/z** | **RT** | **Name** | **FA** | **[M-H_2_O+H]^+^** | **[M-2H_2_O+H]^+^** | **[M+H]^+^ - FA - H_2_O** | **[M+H]^+^-FA-2H_2_O** |
| **CER[NDS]** | 512.4985 | 32.79 | Cer(d18:0/14:0) | 228.0000 | 494.4985 | 476.4985 | 302.4985 | 284.4985 |
|  | 540.5268 | 33.19 | Cer(d18:0/16:0) | 256.0000 | 522.5268 | 504.5268 | 302.5268 | 284.5268 |
|  | 498.4828 | 33.39 | Cer(d18:0/13:0) | 214.0000 | 480.4828 | 462.4828 | 302.4828 | 284.4828 |
|  | 680.6881 | 34.91 | Cer(d18:0/26:0) | 396.0000 | 662.6881 | 644.6881 | 302.6881 | 284.6881 |
|  | 680.6881 | 34.91 | Cer(d20:0/24:0) | 368.0000 | 662.6881 | 644.6881 | 330.6881 | 312.6881 |
|  | 668.6467 | 34.99 | Cer(d18:0/24:0) | 384.0000 | 650.6467 | 632.6467 | 302.6467 | 284.6467 |
|  | 526.5142 | 36.49 | Cer(d18:0/15:0) | 242.0000 | 508.5142 | 490.5142 | 302.5142 | 284.5142 |
|  | 566.5410 | 36.89 | Cer(d18:0/18:1) | 282.0000 | 548.5410 | 530.5410 | 302.5410 | 284.5410 |
|  | 554.5423 | 40.60 | Cer(d18:0/17:0) | 270.0000 | 536.5423 | 518.5423 | 302.5423 | 284.5423 |
| **CER[NS]** | 538.5148 | 32.43 | Cer(d18:1/16:0) | 256.0000 | 520.5148 | 502.5148 | 300.5148 | 282.5148 |
|  | 538.5148 | 32.43 | Cer(d16:1/18:0) | 284.0000 | 520.5148 | 502.5148 | 272.5148 | 254.5148 |
|  | 562.5114 | 33.13 | Cer(d18:2/18:1) | 282.0000 | 544.5114 | 526.5114 | 298.5114 | 280.5114 |
|  | 562.5114 | 33.13 | Cer(d16:2/20:1) | 310.0000 | 544.5114 | 526.5114 | 270.5114 | 252.5114 |
|  | 524.4978 | 33.56 | Cer(d16:1/17:0) | 270.0000 | 506.4978 | 488.4978 | 272.4978 | 254.4978 |
|  | 552.5288 | 34.97 | Cer(d18:1/17:0) | 270.0000 | 534.5288 | 516.5288 | 300.5288 | 282.5288 |
|  | 608.5899 | 35.11 | Cer(d16:1/23:0) | 354.0000 | 590.5899 | 572.5899 | 272.5899 | 254.5899 |
|  | 564.5292 | 35.17 | Cer(d18:1/18:1) | 282.0000 | 546.5292 | 528.5292 | 300.5292 | 282.5292 |
|  | 564.5292 | 35.17 | Cer(d16:1/20:1) | 310.0000 | 546.5292 | 528.5292 | 272.5292 | 254.5292 |
|  | 564.5292 | 35.17 | Cer(d16:2/20:0) | 312.0000 | 546.5292 | 528.5292 | 270.5292 | 252.5292 |
|  | 606.5752 | 35.22 | Cer(d18:2/21:0) | 326.0000 | 588.5752 | 570.5752 | 298.5752 | 280.5752 |
|  | 664.6527 | 35.45 | Cer(d18:1/25:0) | 382.0000 | 646.6527 | 628.6527 | 300.6527 | 282.6527 |
|  | 566.5429 | 36.95 | Cer(d18:1/18:0) | 284.0000 | 548.5429 | 530.5429 | 300.5429 | 282.5429 |
|  | 566.5429 | 36.95 | Cer(d16:1/20:0) | 312.0000 | 548.5429 | 530.5429 | 272.5429 | 254.5429 |
|  | 592.5602 | 38.21 | Cer(d18:2/20:0) | 312.0000 | 574.5602 | 556.5602 | 298.5602 | 280.5602 |
|  | 592.5602 | 38.21 | Cer(d16:1/22:1) | 338.0000 | 574.5602 | 556.5602 | 272.5602 | 254.5602 |
|  | 590.5429 | 38.94 | Cer(d18:2/20:1) | 310.0000 | 572.5429 | 554.5429 | 298.5429 | 280.5429 |
|  | 594.5734 | 41.69 | Cer(d18:1/20:0) | 312.0000 | 576.5734 | 558.5734 | 300.5734 | 282.5734 |
|  | 594.5734 | 41.69 | Cer(d16:1/22:0) | 340.0000 | 576.5734 | 558.5734 | 272.5734 | 254.5734 |
|  | 620.5910 | 42.33 | Cer(d18:2/22:0) | 340.0000 | 602.5910 | 584.5910 | 298.5910 | 280.5910 |
|  | 620.5910 | 42.33 | Cer(d16:2/24:0) | 368.0000 | 602.5910 | 584.5910 | 270.5910 | 252.5910 |
|  | 580.5605 | 43.37 | Cer(d18:1/19:0) | 298.0000 | 562.5605 | 544.5605 | 300.5605 | 282.5605 |
|  | 634.6056 | 44.30 | Cer(d18:2/23:0) | 354.0000 | 616.6056 | 598.6056 | 298.6056 | 280.6056 |
|  | 622.6056 | 44.91 | Cer(d18:1/22:0) | 340.0000 | 604.6056 | 586.6056 | 300.6056 | 282.6056 |
|  | 622.6056 | 44.91 | Cer(d16:1/24:0) | 368.0000 | 604.6056 | 586.6056 | 272.6056 | 254.6056 |
|  | 648.6226 | 45.13 | Cer(d18:1/24:1) | 366.0000 | 630.6226 | 612.6226 | 300.6226 | 282.6226 |
| **CER[NP]** | 556.5210 | 33.19 | Cer(t18:0/16:0) | 256.0000 | 538.5210 | 520.5210 | 318.5210 | 300.5210 |
|  | 584.5545 | 35.13 | Cer(t20:0/16:0) | 256.0000 | 566.5545 | 548.5545 | 346.5545 | 328.5545 |
|  | 612.5858 | 42.11 | Cer(t18:0/20:0) | 312.0000 | 594.5858 | 576.5858 | 318.5858 | 300.5858 |
|  | 612.5858 | 42.11 | Cer(t20:0/18:0) | 284.0000 | 594.5858 | 576.5858 | 346.5858 | 328.5858 |
|  | 668.6471 | 43.52 | Cer(t18:0/24:0) | 368.0000 | 650.6471 | 632.6471 | 318.6471 | 300.6471 |
|  | 668.6471 | 43.52 | Cer(t20:0/22:0) | 340.0000 | 650.6471 | 632.6471 | 346.6471 | 328.6471 |
| **CER[ADS]** | 556.5251 | 30.79 | Cer(d18:0/16:0(2OH)) | 272.0000 | 538.5251 | 520.5251 | 302.5251 | 284.5251 |
|  | 668.6467 | 34.99 | Cer(d18:0/24:0(2OH)) | 384.0000 | 650.6467 | 632.6467 | 302.6467 | 284.6467 |
|  | 668.6467 | 34.99 | Cer(d20:0/22:0(2OH)) | 356.0000 | 650.6467 | 632.6467 | 330.6467 | 312.6467 |
|  | 584.5542 | 35.11 | Cer(d18:0/18:0(2OH)) | 300.0000 | 566.5542 | 548.5542 | 302.5542 | 284.5542 |
|  | 584.5542 | 35.11 | Cer(d20:0/16:0(2OH)) | 272.0000 | 566.5542 | 548.5542 | 330.5542 | 312.5542 |
|  | 612.5870 | 41.46 | Cer(d18:0/20:0(2OH)) | 328.0000 | 594.5870 | 576.5870 | 302.5870 | 284.5870 |
|  | 612.5870 | 41.46 | Cer(d20:0/18:0(2OH)) | 300.0000 | 594.5870 | 576.5870 | 330.5870 | 312.5870 |
| **CER[AS]** | 554.5088 | 31.09 | Cer(d18:1/16:0(2OH)) | 272.0000 | 536.5088 | 518.5088 | 300.5088 | 282.5088 |
|  | 578.5077 | 32.00 | Cer(d16:2/20:1(2OH)) | 326.0000 | 560.5077 | 542.5077 | 270.5077 | 252.5077 |
|  | 580.5255 | 33.33 | Cer(d16:2/20:0(2OH)) | 328.0000 | 562.5255 | 544.5255 | 270.5255 | 252.5255 |
|  | 580.5255 | 33.33 | Cer(d18:2/18:0(2OH)) | 300.0000 | 562.5255 | 544.5255 | 298.5255 | 280.5255 |
|  | 608.5547 | 35.23 | Cer(d16:2/22:0(2OH)) | 356.0000 | 590.5547 | 572.5547 | 270.5547 | 252.5547 |
|  | 608.5547 | 35.23 | Cer(d18:2/20:0(2OH)) | 328.0000 | 590.5547 | 572.5547 | 298.5547 | 280.5547 |
|  | 606.5384 | 35.23 | Cer(d16:2/22:1(2OH)) | 354.0000 | 588.5384 | 570.5384 | 270.5384 | 252.5384 |
|  | 554.5086 | 36.94 | Cer(d18:1/16:0(2OH)) | 272.0000 | 536.5086 | 518.5086 | 300.5086 | 282.5086 |
|  | 550.4782 | 37.83 | Cer(d16:2/18:1(2OH)) | 298.0000 | 532.4782 | 514.4782 | 270.4782 | 252.4782 |
|  | 610.5673 | 39.22 | Cer(d16:1/22:0(2OH)) | 356.0000 | 592.5673 | 574.5673 | 272.5673 | 254.5673 |
|  | 610.5673 | 39.22 | Cer(d18:1/20:0(2OH)) | 328.0000 | 592.5673 | 574.5673 | 300.5673 | 282.5673 |
|  | 636.5857 | 40.52 | Cer(d16:2/24:0(2OH)) | 384.0000 | 618.5857 | 600.5857 | 270.5857 | 252.5857 |
|  | 552.4898 | 41.10 | Cer(d16:1/18:1(2OH)) | 298.0000 | 534.4898 | 516.4898 | 272.4898 | 254.4898 |
|  | 552.4898 | 41.10 | Cer(d16:2/18:0(2OH)) | 300.0000 | 534.4898 | 516.4898 | 270.4898 | 252.4898 |
|  | 578.5089 | 41.77 | Cer(d16:2/20:1(2OH)) | 326.0000 | 560.5089 | 542.5089 | 270.5089 | 252.5089 |
|  | 638.5996 | 43.02 | Cer(d16:1/24:0(2OH)) | 384.0000 | 620.5996 | 602.5996 | 272.5996 | 254.5996 |
|  | 608.5558 | 45.82 | Cer(d16:2/22:0(2OH)) | 356.0000 | 590.5558 | 572.5558 | 270.5558 | 252.5558 |
|  | 608.5558 | 45.82 | Cer(d18:2/20:0(2OH)) | 328.0000 | 590.5558 | 572.5558 | 298.5558 | 280.5558 |
| **CER[AP]** | 740.7023 | 35.06 | Cer(t20:0/26:0(2OH)) | 412.0000 | 722.7023 | 704.7023 | 346.7023 | 328.7023 |
|  | 572.5176 | 36.98 | Cer(t18:0/16:0(2OH)) | 272.0000 | 554.5176 | 536.5176 | 318.5176 | 300.5176 |
|  | 600.5498 | 42.16 | Cer(t18:0/18:0(2OH)) | 300.0000 | 582.5498 | 564.5498 | 318.5498 | 300.5498 |
|  | 600.5498 | 42.16 | Cer(t20:0/16:0(2OH)) | 272.0000 | 582.5498 | 564.5498 | 346.5498 | 328.5498 |
|  | 628.5809 | 44.20 | Cer(t18:0/20:0(2OH)) | 328.0000 | 610.5809 | 592.5809 | 318.5809 | 300.5809 |
|  | 628.5809 | 44.20 | Cer(t20:0/18:0(2OH)) | 300.0000 | 610.5809 | 592.5809 | 346.5809 | 328.5809 |
|  | 656.6115 | 44.45 | Cer(t18:0/22:0(2OH)) | 356.0000 | 638.6115 | 620.6115 | 318.6115 | 300.6115 |
|  | 656.6115 | 44.45 | Cer(t20:0/20:0(2OH)) | 328.0000 | 638.6115 | 620.6115 | 346.6115 | 328.6115 |
| **CER[EOS]** | 832.8034 | 65.08 | 1-O-eicosanoyl-Cer(d18:1/16:0) | 256.0000 | 814.8034 | 796.8034 | 594.8034 | 576.8034 |
|  | 832.8034 | 65.28 | 1-O-stearoyl-Cer(d18:1/18:0) | 284.0000 | 814.8034 | 796.8034 | 566.8034 | 548.8034 |
|  | 748.7098 | 64.50 | 1-O-myristoyl-Cer(d18:1/16:0) | 256.0000 | 730.7098 | 712.7098 | 510.7098 | 492.7098 |


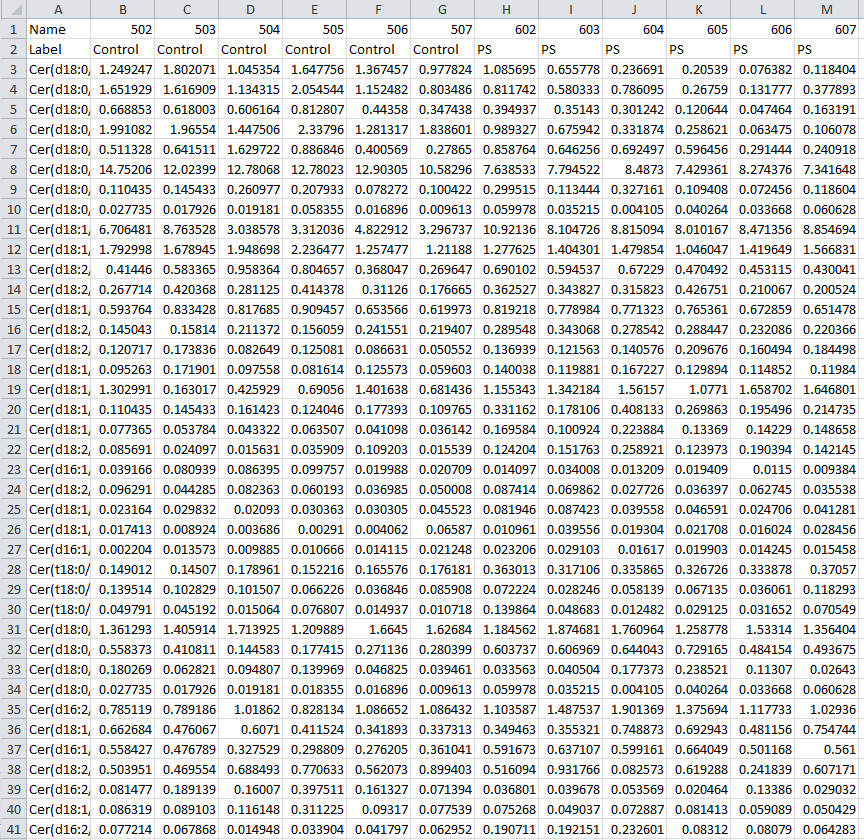
**Table S2**. Peak area of each ceramide molecular species identified in the keratinocytes obtained using MZmine software (XLSX).

**Table S3**. Peak area of each ceramide molecular species identified in the fibroblasts obtained using MZmine software (XLSX).


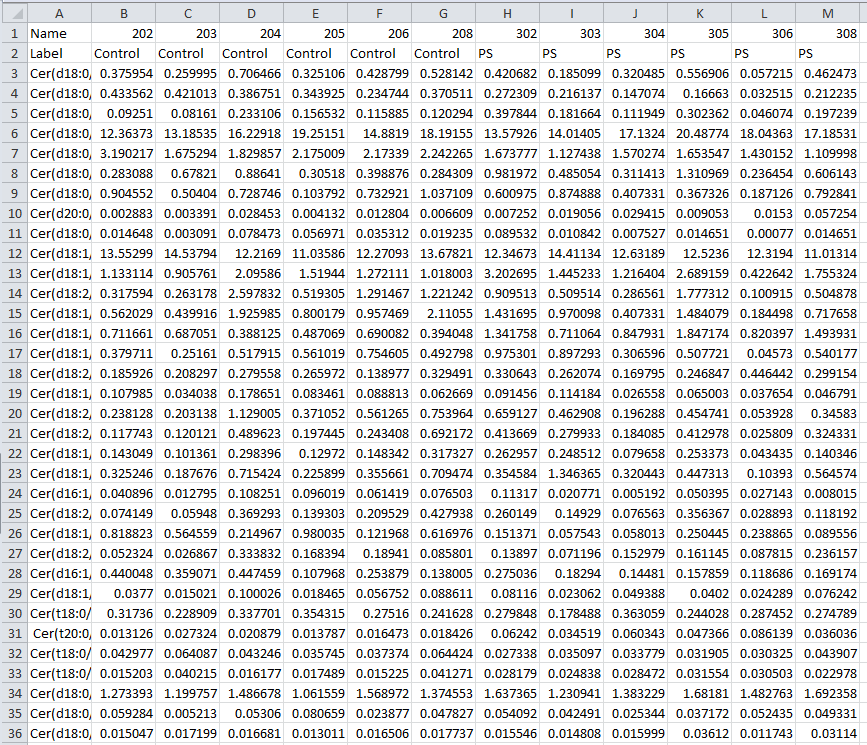

CER[N/A-DS/S/P]

CER[EOS]

**Figure S1.** An example of the total ion chromatogram (TIC).

**Figure S2**. An example of CER specie identification. ESI-MS/MS spectrum of the [M + H]+ ion of Cer(d34:1); [Cer(d18:1/16:0)] [m/z 538.4319; RT 32.43]. Charecteristic fragment ions are indicated with the red boxes.

**Figure S3**. An example of CER specie identification. ESI-MS/MS spectrum of the [M + H]+ ion of Cer(d42:2); [Cer(d18:1/24:1)] [m/z 648.4631; RT 45.13]. Charecteristic fragment ions are indicated with the red boxes.
